# Verified envenomations by crevice weaver spiders (genus *Kukulcania*): Bites are of minor expression but the spiders are commonly misidentified as medically important brown recluses (genus Loxosceles) in North America

**DOI:** 10.1016/j.toxcx.2022.100091

**Published:** 2022-01-19

**Authors:** Richard S. Vetter

**Affiliations:** Department of Entomology, University of California, Riverside, CA, 92521, USA

**Keywords:** Venom, Misidentification, Araneae, Medical aspects, Signs and symptoms

## Abstract

From southern North America, five verified bites by crevice weaver spiders, *Kukulcania* spp. (Filistatidae), are presented here, three of which are pediatric cases. Although the envenomation manifestations were of minimal expression, the salient aspect of this report is that *Kukulcania* spiders are frequently misidentified as brown recluse spiders (genus *Loxosceles*) which are infamous for causing serious dermonecrosis and rarely, life-threatening systemic effects. Misidentification of this relatively harmless spider as a medically important recluse when presented to a physician in an envenomation episode could lead to unwarranted and overzealous treatment such as contraindicated debridement of the affected area.

## Highlights

• Envenomation by *Kukulcania* spiders are of minor concern.
• More importantly is that they are often misidentified as medically important *Loxosceles* spiders.
• Misidentification by medical personnel could result in incorrect and harmful remedies.

## 1 Introduction

Crevice weaver spiders of the genus *Kukulcania* are found throughout the southern third of the United States with many species in Central and South America (Magalhaes and Ramires, 2019). It is a relatively large spider where the males are tan with long spindly legs and small abdomen while in stark juxtaposition, the females are velvety dark brown to black with stocky legs and robust abdomen, looking like small tarantulas. They are common in homes especially in the southeastern United States, hence, their alternate common name is the southern house spider. Therefore, they have higher probability of interacting with a human in an envenomation event than many spider species.

Presented here are five verified envenomations from *Kukulcania* spiders, all with minor manifestation. In each case, the spider was collected at the bite locale immediately after envenomation and it (or a sufficiently diagnostic electronic image in one case) was sent to a qualified arachnologist (i.e., the author) for identification.

## 2 Cases

### 2.1 Case 1

A 17 year-old girl (50 kg, 168 cm height) from southeastern Arizona was bitten on the leg by a male *K. arizonica* while in bed in November. The bite site immediately expressed a white center with pruritus followed by a burning sensation 6–7 hours later. At 24 hours, the bite site was very erythematous, edematous, painful, sore when palpated and warm to the touch. At Day 3, erythema decreased but edema spread. Benadryl was administered with edema lessening. Pruritus and a burning sensation were still apparent through Days 4 and 5. By Day 6, pruritus was waning.

### 2.2 Case 2

A 39 year-old woman (72 kg, 172 cm height) from California's Central Valley stepped on and was bitten on the toe by a male *Kukulcania* spider in August. She had a history of minor allergies and reacted strongly to bee stings as a child. The spider bite felt like a bee sting and within seconds, the bite victim felt a burning sensation. The bee sting pain lasted about 5 minutes, mild burning for about an hour, with a residual white spot at the bite site but with no edema or pruritus.

### 2.3 Case 3

A 5 year-old girl (21 kg, 105 cm height) from the southeastern California desert was bitten on the calf by a large *Kukulcania* spider, either a mature female or late instar immature in May while at school. The child yelled upon being bitten, slapped the spider off her leg whereupon a teacher stepped on the spider and collected it for identification. The bite site was erythematous and the girl scratched the area for 15 minutes while waiting for a parent to pick her up from school. Her parents did not seek medical attention and the girl attended school the next day with no symptoms.

### 2.4 Case 4

A 38 year-old male (no weight or height given) was bitten on the finger by a male *K. hibernalis* in a hotel bed in northwest Georgia in May. The bite was felt immediately but was not painful. The bite victim was being treated for stage II Hodgkin's lymphoma and had a low white blood count. The next morning, the bite site was erythematous, edematous and pruritic. The bite victim was treated with doxycycline and amoxacillin as well as receiving a tetanus shot. The bite healed with minimal noticeability by the 9th day.

### 2.5 Case 5

An 8 year-old boy (29 kg, 134 cm height) was bitten twice on the neck by a male *K. arizonica* in California's southeastern desert in December while toweling off. He had no significant medical history. He developed pruritus and after 10 minutes, had two areas of erythema about 18 mm in diameter with puncture wounds. The bite manifestation was painless and all symptoms resolved in 5 hours.

## 3 Discussion

None of the *Kukulcania* bites produced anything more than minimal self-limiting signs and symptoms of envenomation. Only one bite victim, who had underlying medical issues, contacted a medical authority. Edwards and McCanless (2009) briefly mention two *Kukulcania* bites that manifested in only pain and swelling for 2 days. There is utility in reporting a series of unremarkable, verified spider bites to prevent an exaggerated medical response to harmless or minimally toxic species (e.g., see Isbister and Gray, 2002). Of interest although the sample size is small, two bites occurred in November and December when typically, spider bites are predominantly warm season events (e.g., see Rader et al., 2012).

None of the spiders in the five cases reported here were misidentified because a qualified arachnologist was initially contacted, however, in a study which offered to identify any American spider perceived to be a brown recluse, *Kukulcania* spiders were frequently submitted (122 out of 1773 specimens) (Vetter, 2005). Included in this data set were *Kukulcania* spiders misidentified as *Loxosceles* spiders by authority figures on several occasions: as teaching specimens at a Texas medical school, and by a Georgia physician, a Virginia nurse and pest control technicians in Alabama and California (Vetter, 2009). Misidentification could easily lead to aggressive, unwarranted and overzealous remedy by medical personnel if a *Kukulcania* spider is presented to an attending physician by the bite victim.

### 3.1 How to differentiate Kukulcania from Loxosceles spiders

**Fig. 1:**
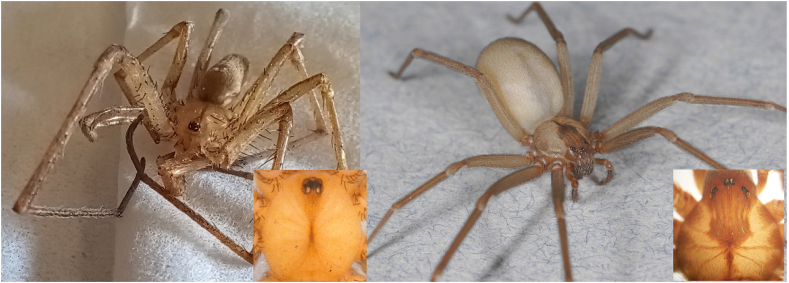
Male *Kukulcania arizonica* from Case 1 (left), permission of photo use given by Matt Sampson. Brown recluse spider (right), photo by R. Vetter. Inserted images of eye patterns are reprinted from *The Brown Recluse Spider*, by Richard S. Vetter. Copyright © 2015 by Cornell University. Used by permission of the publisher, Cornell University Press.

Although male *Kukulcania* spiders share physical similarity to *Loxosceles* spiders, it is very easy to differentiate between the two (Fig. 1). First, eye arrangement: *Kukulcania* spiders have eight eyes all clumped together on a small turret whereas *Loxosceles* spiders have six eyes in pairs in a U-shaped pattern with an anterior pair separated from the lateral pairs by a space. Second, spination: *Kukulcania* spiders have copious, conspicuous spines on their legs whereas *Loxosceles* spiders have no spines on their legs which instead are covered with fine, recumbent hairs.

## Author credits

As a single author paper, I am responsible for it entire content.

## Funding source

None.

## Ethics

All information about bite symptomology was willingly provided voluntarily by the bite victim or the victim's parent and permission to use the information. Geographic location of the envenomations was given broadly in order to increase confidentialty of location.

## Declaration of competing interest

The authors declare that they have no known competing financial interests or personal relationships that could have appeared to influence the work reported in this paper.
